# 4,5,6,7,8,9-Hexahydro-2*H*-cyclo­octa[*c*]pyrazol-1-ium-3-olate

**DOI:** 10.1107/S1600536810043904

**Published:** 2010-10-31

**Authors:** Hoong-Kun Fun, Chin Sing Yeap, R. Venkat Ragavan, V. Vijayakumar, S. Sarveswari

**Affiliations:** a X-ray Crystallography Unit, School of Physics, Universiti Sains Malaysia, 11800 USM, Penang, Malaysia; b Organic Chemistry Division, School of Advanced Sciences, VIT University, Vellore 632 014, India

## Abstract

The title compound, C_9_H_14_N_2_O, exists in the zwitterionic form in the crystal. The cyclo­octane ring adopts a twisted boat-chair conformation. In the crystal, inter­molecular N—H⋯O hydrogen bonds link the mol­ecules into sheets lying parallel to *bc*. The structure is also stabilized by π–π inter­actions, with a centroid-to-centroid distance of 3.5684 (8) Å.

## Related literature

For pyrazole derivatives and their microbial activities, see: Ragavan *et al.* (2009 ▶, 2010 ▶). For a related structure, see: Xiong *et al.* (2007 ▶). For the stability of the temperature controller used for data collection, see: Cosier & Glazer (1986 ▶).
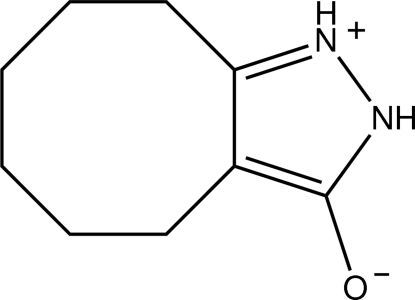


## Experimental

### 

#### Crystal data

C_9_H_14_N_2_O
*M* *_r_* = 166.22
Monoclinic, 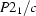

*a* = 12.8078 (2) Å
*b* = 6.7758 (1) Å
*c* = 10.7096 (2) Å
β = 111.620 (1)°
*V* = 864.03 (2) Å^3^
*Z* = 4
Mo *K*α radiation
μ = 0.09 mm^−1^
*T* = 100 K
0.54 × 0.24 × 0.11 mm

#### Data collection

Bruker SMART APEXII CCD area-detector diffractometer
Absorption correction: multi-scan (*SADABS*; Bruker, 2009 ▶) *T*_min_ = 0.956, *T*_max_ = 0.991
6990 measured reflections
1680 independent reflections
1474 reflections with *I* > 2σ(*I*)
*R*_int_ = 0.026

#### Refinement

*R*[*F* ^2^ > 2σ(*F* ^2^)] = 0.037
*wR*(*F* ^2^) = 0.095
*S* = 1.05
1680 reflections
117 parameters
H atoms treated by a mixture of independent and constrained refinement
Δρ_max_ = 0.25 e Å^−3^
Δρ_min_ = −0.25 e Å^−3^

### 

Data collection: *APEX2* (Bruker, 2009 ▶); cell refinement: *SAINT* (Bruker, 2009 ▶); data reduction: *SAINT*; program(s) used to solve structure: *SHELXTL* (Sheldrick, 2008 ▶); program(s) used to refine structure: *SHELXTL*; molecular graphics: *SHELXTL*; software used to prepare material for publication: *SHELXTL* and *PLATON* (Spek, 2009 ▶).

## Supplementary Material

Crystal structure: contains datablocks global, I. DOI: 10.1107/S1600536810043904/fj2356sup1.cif

Structure factors: contains datablocks I. DOI: 10.1107/S1600536810043904/fj2356Isup2.hkl

Additional supplementary materials:  crystallographic information; 3D view; checkCIF report

## Figures and Tables

**Table 1:** Hydrogen-bond geometry (Å, °)

*D*—H⋯*A*	*D*—H	H⋯*A*	*D*⋯*A*	*D*—H⋯*A*
N1—H1N1⋯O1^i^	0.938 (19)	1.757 (19)	2.6900 (14)	173.0 (19)
N2—H1N2⋯O1^ii^	0.925 (19)	1.789 (19)	2.7056 (14)	170.1 (18)

Symmetry codes: (i) 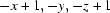; (ii) 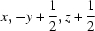.

**Fig. 1.**
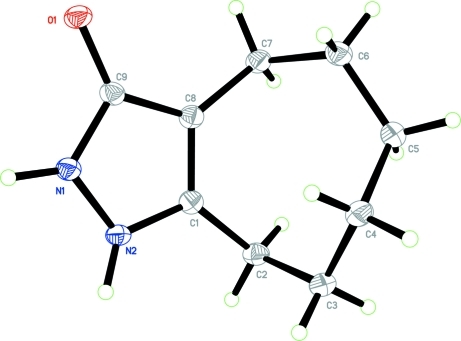
The molecular structure of the title compound with atom labels and 50% probability ellipsoids for non-H atoms.

**Fig. 2.**
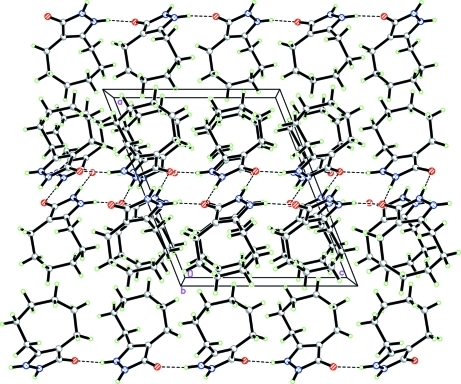
The crystal packing of title compound, viewed down b axis, showing the molecules linked into planes parallel to the bc plane. Intermolecular hydrogen bonds are shown as dashed lines.

## supplementary crystallographic information

### Comment

Antibacterial and antifungal activities of the azoles are most widely studied and some of them are in clinical practice as anti-microbial agents. However, the azole-resistant strains led to the development of new antimicrobial compounds. In particular pyrazole derivatives are extensively studied and used as antimicrobial agents. Pyrazole is an important class of heterocyclic compounds and many pyrazole derivatives are reported to have a broad spectrum of biological properties such as anti-inflammatory, antifungal, herbicidal, anti-tumour, cytotoxic, molecular modelling and antiviral activities. Pyrazole derivatives also act as antiangiogenic agents, A3 adenosine receptor antagonists, neuropeptide YY5 receptor antagonists, kinase inhibitor for treatment of type 2 diabetes, hyperlipidemia, obesity, and thrombopiotinmimetics. Recently urea derivatives of pyrazoles have been reported as potent inhibitors of p38 kinase. Since the high electronegativity of halogens (particularly chlorine and fluorine) in the aromatic part of the drug molecules play an important role in enhancing their biological activity, we are interested to have 4-fluoro or 4-chloro substitution in the aryls of 1,5-diaryl pyrazoles. As part of our on-going research aiming on the synthesis of new antimicrobial compounds, we have reported the synthesis of novel pyrazole derivatives and their microbial activities (Ragavan *et al.*, 2009, 2010).

The title compound exists in an zwitterionic form (Fig. 1). The cyclooctane ring adopts a twisted boat-chair conformation which similar to Xiong *et al.* (2007). In the crystal structure, intermolecular N1—H1N1···O1 and N2—H1N2···O1 hydrogen bonds linked the molecules into planes parallel to the *bc* plane (Fig. 2). The structure is stabilized by the π–π interactions [*Cg*1···*Cg*1^iii^ = 3.5684 (8) Å; *Cg*1 is centroid of N1–N2–C1–C8–C9 ring; (iii) 1 - *x*, 1 - *y*, 1 - *z*].

### Experimental

The compound has been synthesized using the method available in the literature Ragavan *et al.*, (2010) and recrystallized using the ethanol–chloroform 1:1 mixture. Yield: 74%. m.p. 221.6–228.8 °C.

### Refinement

The N-bound H atoms were located from difference Fourier map and refined freely. The rest of H atoms were positioned geometrically [C—H = 0.97 Å] and refined using a riding model [*U*_iso_(H) = 1.2*U*_eq_].

### Crystal data

**Table:**

C_9_H_14_N_2_O	*F*(000) = 360
*M**_r_* = 166.22	*D*_x_ = 1.278 Mg m^−^^3^
Monoclinic, *P*2_1_/*c*	Mo *K*α radiation, λ = 0.71073 Å
Hall symbol: -P 2ybc	Cell parameters from 3802 reflections
*a* = 12.8078 (2) Å	θ = 3.5–30.1°
*b* = 6.7758 (1) Å	µ = 0.09 mm^−^^1^
*c* = 10.7096 (2) Å	*T* = 100 K
β = 111.620 (1)°	Plate, colourless
*V* = 864.03 (2) Å^3^	0.54 × 0.24 × 0.11 mm
*Z* = 4	

### Data collection

**Table:**

Bruker SMART APEXII CCD area-detector diffractometer	1680 independent reflections
Radiation source: fine-focus sealed tube	1474 reflections with *I* > 2σ(*I*)
graphite	*R*_int_ = 0.026
φ and ω scans	θ_max_ = 26.0°, θ_min_ = 1.7°
Absorption correction: multi-scan (*SADABS*; Bruker, 2009)	*h* = −15→15
*T*_min_ = 0.956, *T*_max_ = 0.991	*k* = −8→8
6990 measured reflections	*l* = −13→12

### Refinement

**Table:**

Refinement on *F*^2^	Primary atom site location: structure-invariant direct methods
Least-squares matrix: full	Secondary atom site location: difference Fourier map
*R*[*F*^2^ > 2σ(*F*^2^)] = 0.037	Hydrogen site location: inferred from neighbouring sites
*wR*(*F*^2^) = 0.095	H atoms treated by a mixture of independent and constrained refinement
*S* = 1.05	*w* = 1/[σ^2^(*F*_o_^2^) + (0.047*P*)^2^ + 0.3516*P*] where *P* = (*F*_o_^2^ + 2*F*_c_^2^)/3
1680 reflections	(Δ/σ)_max_ < 0.001
117 parameters	Δρ_max_ = 0.25 e Å^−^^3^
0 restraints	Δρ_min_ = −0.25 e Å^−^^3^

### Special details

**Table:**

Experimental. The crystal was placed in the cold stream of an Oxford Cryosystems Cobra open-flow nitrogen cryostat (Cosier & Glazer, 1986) operating at 100.0 (1) K.
Geometry. All e.s.d.'s (except the e.s.d. in the dihedral angle between two l.s. planes) are estimated using the full covariance matrix. The cell e.s.d.'s are taken into account individually in the estimation of e.s.d.'s in distances, angles and torsion angles; correlations between e.s.d.'s in cell parameters are only used when they are defined by crystal symmetry. An approximate (isotropic) treatment of cell e.s.d.'s is used for estimating e.s.d.'s involving l.s. planes.
Refinement. Refinement of *F*^2^ against ALL reflections. The weighted *R*-factor *wR* and goodness of fit *S* are based on *F*^2^, conventional *R*-factors *R* are based on *F*, with *F* set to zero for negative *F*^2^. The threshold expression of *F*^2^ > σ(*F*^2^) is used only for calculating *R*-factors(gt) *etc*. and is not relevant to the choice of reflections for refinement. *R*-factors based on *F*^2^ are statistically about twice as large as those based on *F*, and *R*- factors based on ALL data will be even larger.

### Fractional atomic coordinates and isotropic or equivalent isotropic displacement parameters (Å^2^)

**Table:**

	*x*	*y*	*z*	*U*_iso_*/*U*_eq_	
O1	0.41579 (8)	0.06094 (13)	0.31883 (8)	0.0190 (2)	
N1	0.44456 (9)	0.21181 (16)	0.52370 (10)	0.0160 (3)	
N2	0.41423 (9)	0.38447 (16)	0.56789 (11)	0.0154 (3)	
C1	0.34257 (10)	0.48051 (18)	0.46046 (12)	0.0149 (3)	
C2	0.28502 (11)	0.66563 (19)	0.47482 (13)	0.0177 (3)	
H2A	0.2711	0.7476	0.3961	0.021*	
H2B	0.3335	0.7384	0.5525	0.021*	
C3	0.17257 (11)	0.6209 (2)	0.49123 (12)	0.0189 (3)	
H3A	0.1883	0.5734	0.5818	0.023*	
H3B	0.1306	0.7430	0.4807	0.023*	
C4	0.09909 (11)	0.4691 (2)	0.39200 (12)	0.0185 (3)	
H4A	0.0295	0.4555	0.4075	0.022*	
H4B	0.1371	0.3426	0.4113	0.022*	
C5	0.07002 (11)	0.5164 (2)	0.24164 (12)	0.0191 (3)	
H5A	−0.0105	0.5025	0.1954	0.023*	
H5B	0.0888	0.6534	0.2339	0.023*	
C6	0.12881 (11)	0.38840 (19)	0.16907 (12)	0.0189 (3)	
H6A	0.0905	0.4065	0.0731	0.023*	
H6B	0.1202	0.2510	0.1889	0.023*	
C7	0.25452 (11)	0.42986 (19)	0.20469 (12)	0.0177 (3)	
H7A	0.2795	0.3581	0.1424	0.021*	
H7B	0.2640	0.5695	0.1918	0.021*	
C8	0.32877 (10)	0.37496 (19)	0.34518 (12)	0.0153 (3)	
C9	0.39626 (10)	0.20329 (18)	0.38724 (12)	0.0150 (3)	
H1N1	0.4969 (14)	0.125 (3)	0.5823 (17)	0.030 (4)*	
H1N2	0.4229 (14)	0.397 (3)	0.6572 (19)	0.037 (5)*	

### Atomic displacement parameters (Å^2^)

**Table:**

	*U*^11^	*U*^22^	*U*^33^	*U*^12^	*U*^13^	*U*^23^
O1	0.0272 (5)	0.0181 (5)	0.0123 (4)	0.0062 (4)	0.0082 (4)	0.0006 (3)
N1	0.0201 (6)	0.0158 (5)	0.0122 (5)	0.0031 (4)	0.0061 (4)	0.0007 (4)
N2	0.0181 (6)	0.0167 (5)	0.0122 (5)	0.0012 (4)	0.0065 (4)	−0.0012 (4)
C1	0.0149 (6)	0.0163 (6)	0.0150 (6)	−0.0015 (5)	0.0072 (5)	0.0013 (5)
C2	0.0214 (7)	0.0155 (6)	0.0166 (6)	−0.0001 (5)	0.0076 (5)	−0.0017 (5)
C3	0.0204 (7)	0.0207 (7)	0.0165 (6)	0.0025 (5)	0.0078 (5)	−0.0021 (5)
C4	0.0179 (7)	0.0212 (7)	0.0182 (6)	0.0003 (5)	0.0090 (5)	−0.0014 (5)
C5	0.0179 (7)	0.0217 (7)	0.0163 (7)	0.0025 (5)	0.0045 (5)	−0.0006 (5)
C6	0.0223 (7)	0.0203 (7)	0.0130 (6)	0.0034 (5)	0.0051 (5)	0.0004 (5)
C7	0.0227 (7)	0.0192 (6)	0.0126 (6)	0.0053 (5)	0.0082 (5)	0.0035 (5)
C8	0.0165 (6)	0.0168 (6)	0.0144 (6)	0.0001 (5)	0.0078 (5)	0.0012 (5)
C9	0.0168 (6)	0.0172 (6)	0.0118 (6)	−0.0001 (5)	0.0061 (5)	0.0011 (5)

### Geometric parameters (Å, °)

**Table:**

O1—C9	1.2902 (15)	C4—C5	1.5467 (17)
N1—C9	1.3622 (16)	C4—H4A	0.9700
N1—N2	1.3708 (15)	C4—H4B	0.9700
N1—H1N1	0.939 (18)	C5—C6	1.5343 (17)
N2—C1	1.3459 (16)	C5—H5A	0.9700
N2—H1N2	0.925 (19)	C5—H5B	0.9700
C1—C8	1.3807 (17)	C6—C7	1.5377 (18)
C1—C2	1.4912 (17)	C6—H6A	0.9700
C2—C3	1.5438 (17)	C6—H6B	0.9700
C2—H2A	0.9700	C7—C8	1.5007 (17)
C2—H2B	0.9700	C7—H7A	0.9700
C3—C4	1.5283 (18)	C7—H7B	0.9700
C3—H3A	0.9700	C8—C9	1.4199 (17)
C3—H3B	0.9700		
			
C9—N1—N2	109.39 (10)	H4A—C4—H4B	107.4
C9—N1—H1N1	128.4 (10)	C6—C5—C4	115.83 (11)
N2—N1—H1N1	121.9 (10)	C6—C5—H5A	108.3
C1—N2—N1	107.96 (10)	C4—C5—H5A	108.3
C1—N2—H1N2	128.7 (11)	C6—C5—H5B	108.3
N1—N2—H1N2	119.5 (11)	C4—C5—H5B	108.3
N2—C1—C8	109.65 (11)	H5A—C5—H5B	107.4
N2—C1—C2	121.73 (11)	C5—C6—C7	115.82 (11)
C8—C1—C2	128.51 (11)	C5—C6—H6A	108.3
C1—C2—C3	111.34 (10)	C7—C6—H6A	108.3
C1—C2—H2A	109.4	C5—C6—H6B	108.3
C3—C2—H2A	109.4	C7—C6—H6B	108.3
C1—C2—H2B	109.4	H6A—C6—H6B	107.4
C3—C2—H2B	109.4	C8—C7—C6	114.98 (10)
H2A—C2—H2B	108.0	C8—C7—H7A	108.5
C4—C3—C2	114.47 (10)	C6—C7—H7A	108.5
C4—C3—H3A	108.6	C8—C7—H7B	108.5
C2—C3—H3A	108.6	C6—C7—H7B	108.5
C4—C3—H3B	108.6	H7A—C7—H7B	107.5
C2—C3—H3B	108.6	C1—C8—C9	106.16 (11)
H3A—C3—H3B	107.6	C1—C8—C7	126.38 (11)
C3—C4—C5	115.79 (11)	C9—C8—C7	127.44 (11)
C3—C4—H4A	108.3	O1—C9—N1	122.31 (11)
C5—C4—H4A	108.3	O1—C9—C8	130.92 (11)
C3—C4—H4B	108.3	N1—C9—C8	106.76 (11)
C5—C4—H4B	108.3		
			
C9—N1—N2—C1	2.98 (13)	C2—C1—C8—C9	−175.54 (12)
N1—N2—C1—C8	−2.13 (13)	N2—C1—C8—C7	178.99 (11)
N1—N2—C1—C2	174.25 (11)	C2—C1—C8—C7	2.9 (2)
N2—C1—C2—C3	−89.09 (14)	C6—C7—C8—C1	−77.60 (16)
C8—C1—C2—C3	86.55 (15)	C6—C7—C8—C9	100.55 (14)
C1—C2—C3—C4	−46.15 (14)	N2—N1—C9—O1	176.18 (11)
C2—C3—C4—C5	−55.64 (15)	N2—N1—C9—C8	−2.61 (13)
C3—C4—C5—C6	108.08 (13)	C1—C8—C9—O1	−177.37 (13)
C4—C5—C6—C7	−72.87 (15)	C7—C8—C9—O1	4.2 (2)
C5—C6—C7—C8	68.15 (15)	C1—C8—C9—N1	1.29 (13)
N2—C1—C8—C9	0.52 (14)	C7—C8—C9—N1	−177.16 (11)

### Hydrogen-bond geometry (Å, °)

**Table:**

*D*—H···*A*	*D*—H	H···*A*	*D*···*A*	*D*—H···*A*
N1—H1N1···O1^i^	0.938 (19)	1.757 (19)	2.6900 (14)	173.0 (19)
N2—H1N2···O1^ii^	0.925 (19)	1.789 (19)	2.7056 (14)	170.1 (18)

Symmetry codes: (i) −*x*+1, −*y*, −*z*+1; (ii) *x*, −*y*+1/2, *z*+1/2.
